# Animal Models of Psychiatric Disorders and Their Relevance to Alcoholism

**Published:** 2000

**Authors:** Robert Hitzemann

**Affiliations:** Robert Hitzemann, Ph.D., is a professor and chairman of the Department of Behavioral Neuroscience, Oregon Health Sciences University, Portland, Oregon, and a research pharmacologist at the Research Service, Veterans Affairs Medical Center, Portland, Oregon

**Keywords:** animal model, behavioral and mental disorder, AOD (alcohol or other drug) dependence, comorbidity, validity (research methods), reliability (research methods), schizophrenia, emotional and psychiatric depression, fear, anxiety, animal strains

## Abstract

Animal models are important tools in the study of psychiatric disorders, including alcoholism,^1^ because they allow the use of research methods that cannot be used for ethical reasons in humans. Consequently, scientists have developed numerous approaches to evaluate the validity and reliability of animal models for studying human behavior and human disorders. Researchers have developed animal models of schizophrenia, fear and anxiety, depression, and alcoholism, all of which are being used to study the relationship between alcoholism and co-occurring psychiatric disorders. These models may help researchers and clinicians determine how best to treat patients with alcoholism and co-occurring psychiatric disorders.

Scientists are increasingly being asked both to develop and to defend the use of animal models for studying psychiatric disorders that appear to be uniquely human, such as alcohol and other substance abuse, schizophrenia, depression, and anxiety. These disorders either are not observed in animals or cannot be directly measured (i.e., the animals cannot verbally indicate what they think or feel). This review examines some of the animal models used to study these disorders, with a special emphasis on the use of these models in the search for genes that contribute to the disorders.

The general argument for using animal models in behavioral research is that such models allow researchers to test specific hypotheses under highly controlled conditions using methods that are either impossible or unethical to use in humans. For example, researchers can create genetically altered mice (e.g., mice with a foreign gene added to their genetic makeup) to examine the influence of specific gene products on behavior. Recent advances in genetic engineering allow investigators to activate or inactivate specific genes in specific regions of the brain. Other research techniques that scientists can only use in animals include injecting materials directly into the brain and implanting brain electrodes.

Critics of animal models in behavioral research have argued that these studies could be conducted using animals with simpler systems, such as worms, or computer simulations. This argument is based on the theory that the basic processes that increase or decrease the strength of communication between and among cells (i.e., the synaptic connections), and therefore influence behavior, are probably similar in organisms as “simple” as the worm and as “complex” as the human. In higher vertebrates, however, these basic processes combine into highly sophisticated and complex arrays that no computer system has yet been able to duplicate. Thus, throughout the foreseeable future, researchers investigating complex behavioral traits and characteristics (i.e., phenotypes) of humans will continue to be forced to use animals with well-developed central nervous systems rather than simpler organisms or computer models. Although rodents are not as appropriate as are primates for modeling all aspects of a specific human behavior, rodents will carry the main workload of animal research for some time to come.

This article describes animal models of schizophrenia, fear and anxiety, and depression. It also discusses the use of animal models in studying alcoholism and co-occurring psychiatric disorders. First, however, validity and reliability—the criteria used to evaluate animal models—are defined.

## Criteria for Animal Models of Human Behavior

Validity and reliability are the main criteria for evaluating animal models (for a review, see Geyer and Markou 1995). This article first examines the four aspects of validity—face, predictive, etiological, and genetic—and then focuses on the issue of reliability.

*Face validity* refers to the similarity between the animal model and the specific human behavior of interest. Superficially, it would seem that the animal behavior should mimic the related human behavior as much as possible. For example, mouse and man display similar responses to a loud noise (i.e., startle responses). Both respond with an eye-blink, a rapid change in heart rate, and some form of whole body movement (e.g., a mouse jumps, a person jerks). However, when studying the regulation of the startle response, the observed response need not be obviously similar. For investigative purposes, the results would be just as informative if the mouse yawned or curled its tail, providing that these phenotypes could be reliably measured and had predictive, etiological, and genetic validity (described later in this article).

Researchers studying animal models of human psychiatric disorders are increasingly examining intermediate phenotypes or endophenotypes (i.e., markers that may indicate a susceptibility to a disorder). Endophenotypes generally are not immediately visible, but they may contribute to the susceptibility to develop a particular behavior or syndrome. The use of endophenotypes is helpful in that the underlying neurobiology (i.e., the mechanisms or mechanism by which the endophenotype increases susceptibility) is frequently known and thus researchers have the advantage (i.e., in the case of genetic studies) of linking specific gene(s) that may influence the behavior with specific brain mechanisms. A disadvantage of endophenotypes, however, is their lack of specificity. For example, prepulse inhibition of the acoustic startle response (ASR)^2^ serves as an operational measure, or endophenotype, for the deficits in the mechanisms that allow normal individuals to filter or block most of the sensory and cognitive stimuli that they receive (i.e., sensory gating abnormalities). Such abnormalities are often seen in schizophrenia (see table 1). However, whereas schizophrenics show poor prepulse inhibition, deficits also are seen in Tourette’s syndrome, bed-wetting incidents, and during the menstrual cycle.

Furthermore, the popularity of endophenotypes may derive partly from the fact that they generally have face validity (i.e., a complex human behavior has been reduced to something that can be measured under similar conditions in laboratory animals and humans). However, as previously stated, face validity for an endophenotype does not justify its use.

*Predictive validity* refers to the extent that an animal model can allow a researcher to make predictions about the human phenotype. In many cases, predictive validity refers to how useful animal models are for predicting the efficacy and safety of drugs for treating psychiatric disorders. For example, new antipsychotic drugs were screened for years by their ability to block (in rodents) amphetamine-induced behaviors (e.g., increased licking, gnawing, and chewing). This test was based on the hypothesis that because amphetamine increases levels of dopamine (a key brain chemical involved in nerve cell communication) and psychosis is associated with increased levels of dopamine, drugs that block amphetamine-induced behavior would be effective as antipsychotics.

However, the drugs selected using this test also produced extra-pyramidal symptoms (EPS), such as drug-induced Parkinsonism. EPS was one of the major reasons that people did not comply with drug treatment. This problem eventually led to the development of new atypical antipsychotic drugs (i.e., drugs that maintain antipsychotic efficacy but do not produce EPS). New drugs were screened for a particular receptor profile (i.e., either high or low affinity for a particular receptor—that is, a protein that binds to a specific brain chemical, or neurotransmitter). Once these compounds were identified, the amphetamine test was used to determine if the new compounds could block the drug-induced behaviors by a novel mechanism. Screens such as this led to the development of a new generation of atypical antipsychotics (e.g., risperidone and olanzepine).

*Etiological validity* exists when one can determine—to the extent possible—that the mechanisms involved in a behavior observed in the animal model are similar to those involved in the associated human behavior. Historically, researchers have established etiological validity by using the animal model to determine the underlying mechanisms of behavior and then by testing the role of such mechanisms in human behavior. In coming years, however, the reverse process probably will also be increasingly used: researchers will first examine the underlying mechanisms in humans and then examine animal models to determine whether similar mechanisms are at work.

For example, in a group of normal human controls, Volkow and colleagues (1999) found that human subjects with naturally low D_2_ dopamine receptor density reported a euphoric effect in response to an intravenous injection of methylphenidate (Ritalin^™^). These data led Thanos and colleagues (1999) to investigate whether elevating D_2_ receptor density in rats would reduce alcohol preference. An increased receptor density was associated with reduced alcohol preference. Thus, the researchers would test the relationship between receptor density and drug preference under controlled laboratory conditions.

*Genetic validity* exists when the risk for a disease is known to have a similar genetic component in both humans and the animal model. At least 50 percent of the risk for psychiatric disorders such as schizophrenia, depression, and alcoholism can be attributed to inherited genetic factors. Thus, for any animal model of these disorders, it would be useful—but not necessary—to demonstrate a significant genetic component to the natural phenotypical variation. Various strategies can be used, with the eventual goal of detecting relevant genes. Researchers have made considerable progress in this area for behavioral phenotypes (Crabbe et al. 1999), although results still lag behind the detection of genes for other complex phenotypes (e.g., diabetes, obesity, and hypertension) (e.g., Pomp 1997).

### Reliability of the Model

The reliability of the animal model refers to the stability and reproducibility of the phenotype. Some animal phenotypes appear to show remarkable stability. For example, by using alcohol-induced locomotion as a measure of alcohol’s acute stimulant response^3^ in a series of inbred mouse strains (Demarest et al. 1999), we obtained results essentially identical to those obtained by Crabbe and colleagues (1983).

Stability of the phenotype also can refer to test-retest reliability, a construct frequently used in studies on humans. Scientists working with animals, however, have been reluctant to use a test-retest design; arguing—and frequently demonstrating—that the first test will significantly affect the second test. When working with inbred (i.e., genetically identical) animal strains, the extent to which this is a problem can be measured easily by comparing animals tested once with animals tested multiple times. However, when using genetically segregating populations (i.e., in which each individual is genetically unique), such a solution is not possible. In order to make a sound decision, researchers must weigh the advantages and disadvantages of repeatedly testing animals. Because the main effect of poor test-retest reliability is to decrease statistical power, increasing sample size and testing only once is a solution to the problem.

### Suitability of the Animal Species for a Specific Experimental Need

Historically, behavioral studies have largely relied on the use of the albino outbred rat (i.e., each animal is genetically unique) as the primary experimental species. For genetic studies, however, mice offer advantages over rats, such as the availability of numerous inbred mouse strains, animals in which genes have been added or deleted (i.e., transgenic animals), and a well-described genome. When rat paradigms are adapted to the mouse, especially paradigms involving higher order learning, mice tend to do poorly (Crawley et al. 1997). Whether or not this difference reflects a difference in “intelligence” or the need for new protocols specifically suited to the mouse’s abilities and temperament is unclear.

Mice present other disadvantages to researchers as well. For example, researchers frequently encounter marked sensory deficits when using inbred mouse strains. Numerous strains, including some of those most frequently used (e.g., DBA/2J, C57BL/6J, and BALB/cJ) have marked high frequency hearing deficits, which in the DBA/2J can be detected as early as 4 to 6 weeks of age. Other strains (e.g., the C3H/HeJ) show marked retinal degeneration at an early age. Obviously, such animals would be unsuitable for tests that require the animal to remember spatial cues. In addition, albino rats and mice show impaired vision under bright lights, which they are exposed to in the standard open-field test (see table 1), a deficit that must be accounted for when interpreting their performance. Although the availability of transgenic mice is an advantage for research, this technology presents disadvantages as well (see the article in this issue by Bowers, pp. 175–184).

## Models of Psychiatric Disorders

Investigators use animal models to study the mechanisms involved in several psychiatric disorders and to investigate the relationship between alcoholism and co-occurring disorders. This section describes animal models for schizophrenia, depression, and fear and anxiety. The use of animal models to study alcoholism and co-occurring psychiatric disorders is discussed in the subsequent section.

### Schizophrenia

Schizophrenia is one of the most disabling of all psychiatric disorders. The lifetime risk of developing schizophrenia is approximately 1 percent, a risk rate that appears to be largely independent of race, culture, and socioeconomic status. Schizophrenia is characterized by both positive symptoms (e.g., delusions, hallucinations, and thought disorder) and negative symptoms (e.g., withdrawal and a dementia-like state). In addition, the mechanisms of schizophrenia appear to be highly diverse. Given this level of complexity, the difficulty in developing an animal model that can capture the schizophrenic pathology is apparent. Progress has been made in this effort, and a few of the approaches used are described here.

Schizophrenia is characterized by deficits in attention-related processes, information processing, and the sensing and filtering (i.e., sensorimotor gating) of environmental stimuli (Braff 1993). Prepulse inhibition of the acoustic startle response (described in table 1) has been used to measure the deficits in sensorimotor gating associated with schizophrenia. The data obtained led to increased interest in the related animal model, an approach that has several advantages. Measurements of prepulse inhibition are easily adapted to mice and rats, and the underlying mechanisms regulating prepulse inhibition appear to be similar, if not identical, in animals and humans. Antipsychotic drugs improve poor prepulse inhibition, and dopamine agonists (i.e., compounds that combine with dopamine receptors) decrease prepulse inhibition. Finally, the regulation of prepulse inhibition in animals is markedly influenced by genetic factors. Thus, prepulse inhibition has face, predictive, construct, and genetic validity.

In addition to prepulse inhibition, poor latent inhibition is also associated with schizophrenia (Braff 1993) and can be easily measured in animals. Latent inhibition refers to the inhibition of a conditioned response as a result of pre-exposure to the conditioned stimulus. In our laboratory, we have measured latent inhibition in mice as follows. Mice were randomly divided into two groups. One group was given 50 trials, each consisting of a 10-second conditioning stimulus (CS) (i.e., a 2,000 hertz, 85 decibel tone); each trial was separated by 5 to 75 seconds. The second group (i.e., the control group) did not receive the auditory CS. On the second day, both groups were assessed for the acquisition of a conditioned avoidance response (CAR) in which the CS was associated with a mild footshock. Success on the test was measured by how quickly the animals learned to avoid the mild shock. Latent inhibition was observed in the preexposed group (i.e., they acquired the CAR more slowly).

Considerable evidence suggests that dopamine has a regulatory role in both prepulse inhibition of the acoustic startle response and latent inhibition (reviewed in Kline and colleagues 1998). These data led us to investigate prepulse inhibition and latent inhibition in lines of mice that had been selectively bred to respond or not respond to the drug haloperidol, a typical antipsychotic (also termed neuroleptic). Catalepsy, the mouse equivalent to extra pyramidal symptoms, was used to measure response to the drug (see table 1). Two lines were created, neuroleptic responsive (NR) and neuroleptic nonresponsive (NNR), which differ about twentyfold in their sensitivity to haloperidol. Numerous differences have been observed in brain dopamine systems between the NR and NNR lines, including the number of dopamine neurons and the density of D_2_ dopamine receptors (Hitzemann et al. 1995).

Given these differences, unsurprisingly, Kline and colleagues (1998) found marked deficits in both prepulse inhibition and latent inhibition in the NNR mice when compared with the NR line. From these data, we can conclude the following, at least in mice: (1) some of the same genes regulating the catalepsy response also regulate pre-pulse inhibition and latent inhibition, and (2) elucidating these genes will likely be important to understanding the underlying mechanisms of these behaviors. The question of whether or not such studies will illuminate schizophrenic pathology remains unclear. However, the information obtained provides new starting points for future investigations—starting points that would not have been possible without the use of animal studies.

In addition to the endophenotype approach, researchers have used other strategies to model aspects of schizophrenia, including administering hallucinogens and acute and chronic administration of stimulants (e.g., amphetamine). A number of problems occur with these approaches (see Geyer and Markou 1995). Because hallucinations cannot be observed in mice and rats, researchers have used endpoints, such as behavioral disruption or locomotor stimulation (many hallucinogens are also stimulants). Furthermore, the hallucinations induced by drugs like LSD are primarily visual, whereas those seen in schizophrenia are more likely to be auditory or olfactory.

Stimulant models of psychosis arose after reports that stimulant abuse resulted in paranoid delusions. Researchers conducted controlled chronic administration studies to demonstrate that the paranoia was not associated with a pre-existing psychiatric disorder but resulted from a maladaptive drug response. The chronic administration of stimulants to most strains of rats and to some strains of mice leads to drug sensitization (i.e., increased sensitivity) characterized by an increase in certain behaviors (e.g., patterned locomotion, highly repetitive grooming, and gnawing and licking movements). Thus, researchers developed an animal model in which increased motor responses substituted for the paranoid delusions observed clinically. Despite the lack of face validity, the argument was made that the model was likely to have etiological validity. Furthermore, the stimulant sensitization model both developed from and added to the hypothesis that psychosis is related to excesses in dopamine activity. In recent years, researchers investigating psychosis and schizophrenia have favored other theories over the dopamine hypothesis. However, the sensitization model has been adopted by alcohol and other drug abuse researchers to investigate the adaptive and pathological effects of chronic intoxication. The validity and heuristic value of this approach is detailed in this issue and in volume 24, number 2.

Another approach to modeling schizophrenia, which has elements of both the hallucinogen and stimulant strategies, examines drugs that act by blocking receptors for *N*-methyl-d-aspartate (NMDA) (i.e., NMDA antagonists), such as phencyclidine (PCP), ketamine, and MK-801. Soon after the introduction of PCP in the 1950s, researchers recognized that the drug could produce both positive and negative symptoms of schizophrenia in normal control subjects and intensify psychotic symptoms in chronic schizophrenics. Animal models developed from these observations have largely focused on NMDA antagonist-induced increases in locomotor activity and disruption of sensory gating (e.g., prepulse inhibition of the acoustic startle response) (Geyer and Markou 1995).

### Depression

Depression is a common disorder (i.e., the lifetime prevalence is 15 to 25 percent) and is frequently encountered both in the psychiatric clinic and the primary care setting. In addition to symptoms such as depressed mood, failing to find pleasure from normally pleasurable activities, and feelings of worthlessness and guilt, typical depression is characterized by a number of vegetative signs, including slowed activity, insomnia (or hypersomnia), and weight loss (or gain).

One of the earliest models of depression was built from the clinical observation that more than 10 percent of patients taking the antihypertension drug reserpine develop some symptoms of depression. In animals, reserpine causes locomotor depression. The observation that the locomotor depression could be reversed by 5-hydroxytryptophan (5–HTP) (a precursor to serotonin) or dihydroxyphenylserine (DOPS) (a precursor of norepinephrine) led to the development of the theory that depression occurred as a result of a deficiency in serotonin or norepinephrine (a theory that has changed little in more than 40 years). Researchers have expanded the theory based on the observations that inhibitors of the enzyme cholinesterase caused clinical symptoms of depression and psychomotor depression in animals. Thus, depression was thought to develop from an imbalance between high cholinergic activity on one side and low serotonergic or noradrenergic activity on the other.

Learned helplessness and the related phenotypes of behavioral despair and chronic unpredictable stress have been used to model some aspects of depression. Martin Seligman originally developed the learned helplessness model by exposing dogs to shocks from which they could not escape. The dogs eventually gave up and made no effort to escape the shocks (i.e., they became helpless). In addition, other behaviors were affected (e.g., the dogs appeared apathetic and had poor appetites).

The importance of the learned helplessness model in understanding the pathology of depression builds from the argument that helplessness is a common characteristic of depression and that depression can improve if the clinician instills in the patient a sense of mastery and control over the environment. Rather than debating the value of this model (if any) to understanding depression, we will discuss the observance of learned helplessness in animals. The model has several problems. For example, only a relatively small proportion of the animals exposed to the inescapable shock become truly helpless. Among outbred stocks of rats, such as the Sprague-Dawley or Wistar, only 10 to 50 percent develop the helplessness syndrome (Willner 1991). The low incidence may be attributable to genetic factors, but the data also suggest that the phenomenon is not a common adaptive mechanism. Furthermore, among inbred strains of mice, some will exhibit helplessness in the absence of training (i.e., the B6 mouse freezes in response to shock and does not attempt to escape). These data suggest that some of the differences between helpless and nonhelpless animals may relate to natural differences in the pattern of motor responses. Furthermore, other investigators have emphasized the difficulty in unraveling helplessness from a shock-induced reduction in motor activity (see Willner 1991), which is commonly seen in rodents in response to any chronic unpredictable stress (e.g., crowding, irregular feeding, and interrupted sleep). In contrast with these concerns, the model has had reasonably good predictive validity from a psychopharmacological perspective; a wide variety of antidepressants and electroconvulsive shocks reduce the helpless behavior.

Behavioral despair is related conceptually to learned helplessness. In this model, researchers forced rodents to swim in a tank from which they could not escape. At the outset, both the mice and rats swam vigorously. Over time, however, the animals become immobile, a condition Porsalt (1981) called behavioral despair. The underlying construct validity of this model was questionable (e.g., the immobility could simply have been an efficient coping strategy). However, the model had high predictive validity in identifying antidepressants, especially after chronic administration (Willner 1991). Thus, the swim test and modifications of it (e.g., coupling the test with chronic unpredictable stress) were well-used models.

### Fear and Anxiety

Both fear and anxiety are alerting signals that warn the individual against impending danger and enable the individual to take defensive measures. For animals, the distinctions between fear and anxiety are vague. For the purposes of this discussion, the animal models are divided into those that measure conditioned fear (i.e., fear that is learned by association with an aversive stimulus) and unconditioned fear (i.e., fear that is not learned). Unconditioned fear is one of the behavioral components measured in the standard open-field test, one of the most reliable measures in behavioral science. The test, which has been developed and refined over the past 70 years (Hall 1934), consists of placing the animal in a brightly lit novel environment in which it cannot return to the home cage. The novel environment and the light provide the stressors, because rodents are photophobic. Increased emotionality and anxiety is associated with a decrease in locomotor activity and rearing, increased activity in the periphery versus the central part of the testing area (thigmotaxis), and defecation (see table 1).

Researchers also measure unconditioned fear and anxiety in the light-dark transition test and in the elevated plus maze, both of which present approach-avoidance conflicts. The light-dark transition test is a modification of the standard open-field test in which the animal is provided a dark enclosure in the open-field environment. The animal must choose between its natural tendency to explore the open field with the tendency to escape from the bright light. The elevated plus maze presents the animal with a similar conflict; the animal must choose between the tendency to explore a novel environment and the tendency to escape from the elevated open arms of the maze. (See table 1 for further description of these tests.) For both tests, anxiety-reducing drugs increase exploratory activity (Crawley et al. 1997). However, when the researchers compared the genetically identical mice based on the two tests, the correlations between the two tests on both baseline activity and drug-induced activity were weak, suggesting that different mechanisms were involved.

Conditioned fear, which combines elements of learning and memory with fear demonstration, has been studied in several contexts. Fear-potentiated startle (FPS) has been extensively studied in rats (Davis et al. 1997) and more recently in mice (Falls et al. 1997; McCaughran et al. 2000). In the basic paradigm, animals are trained on day 1 to associate the conditioned stimulus (CS) (e.g., a light or sound cue) with the unconditioned stimulus (US) (e.g., foot shock). On the second day, the animals are placed in the acoustic startle apparatus (i.e., a device that measures acoustic startle response). The startle response is then measured in the presence and absence of the CS. The degree of conditioned fear is measured by the increase in the degree of the acoustic startle response when the CS is present. In humans, the acoustic startle response is enhanced in anticipation of aversive shock and in posttraumatic stress disorder (see McCaughran et al. 2000). In rats, the FPS model appears to have good predictive validity in that the startle response is weakened by anxiety-reducing drugs, such as diazepam, and enhanced by anxiety-inducing drugs, such as yohimbine. The rat model also appears to have high construct validity (see Davis et al. 1997), and researchers have described the neuronal mechanisms that regulate FPS.

Compared with rats, the situation in mice appears to be significantly more complicated. Falls and colleagues (1997) found that a marked FPS response could be detected in the DBA/2J (D2) but not in the C57BL/6J (B6) inbred strain. This finding was confirmed by McCaughran and colleagues (2000). These data were of interest, because in a contextual fear-conditioning paradigm, in which the animal is trained to associate the CS with a US in a particular environment (i.e., the context), the B6 and many other inbred strains perform quite well and the D2 performs poorly (Crawley et al. 1997; Owen et al. 1997). On a cued paradigm, in which the conditioned fear response is measured by bouts of freezing, the B6 and D2 have essentially identical responses (Owen et al. 1997). Thus, depending on the conditioning paradigm used, the B6 response is greater than, less than, or equal to the D2 response. It is important to recognize that such differences exist and that these differences must be kept in mind as one extends from the animal model to the clinic.

## Models of Psychiatric Disease and Alcoholism

Animal models of alcoholism have been discussed elsewhere in this issue and in volume 24, number 2. Thus, this article focuses on models that integrate alcoholism and other psychiatric diseases and describes some examples of such models. The significance of the models is attributable to the high comorbidity of alcoholism with other disorders. The most common comorbid psychiatric diagnoses with alcoholism are other substance abuse-related disorders, antisocial personality disorder, mood disorders, and anxiety disorders. The question of which disorder arises first remains controversial. Animal models offer the advantage of allowing researchers to test the interaction from both directions under highly controlled conditions. Furthermore, the issues being addressed go beyond underlying mechanisms of the disorders to the development of new treatment modalities. Thirty to forty percent of people diagnosed with alcohol abuse or dependence are likely to develop a major depressive episode during their lifetime (two to three times the normal incidence). Twenty-five to fifty percent of people with alcohol use disorders also meet the criteria for anxiety disorders. Research with animal models may help determine which treatments are most effective in reducing alcohol consumption among people with an underlying psychiatric disorder, if in fact this can be determined with animal models.

Researchers are accumulating data to address some of these issues. For the BXD recombinant inbred (RI) series^4^ of mice, data have been collected on alcohol consumption, preference, and acceptance (Phillips et al. 1994; Rodriquez et al. 1994); open-field activity (Koyner et al. 2000); FPS (McCaughran et al. 2000); context fear conditioning (Owen et al. 1997); and cued fear conditioning (Owen et al. 1997). The cumulative nature of the RI data allows investigation into whether genetic relationships exist between the alcohol phenotypes and the animal models of fear and anxiety. The results are summarized in table 2 and illustrate that no significant correlations exist between the alcohol phenotypes and the fear and anxiety phenotypes. Thus, we conclude that the genes that predispose the BXD RI animals to high alcohol consumption (i.e., a major feature of alcoholism) are not the same genes that predispose the animal to demonstrate high levels of unconditioned and conditioned fear and anxiety. However, these results should be viewed cautiously. The results described in table 2 refer only to this particular RI series, derived from the B6 and D2 inbred mouse strains. Although the B6 and D2 strains are highly polymorphic and differ markedly in a number of behavioral phenotypes, the data obtained are valid only for understanding the differences between the B6 and D2 strains.

Recent findings (Hitzemann et al. in press) show that in crosses derived from other inbred strains, the genes associated with alcohol phenotypes are different from those detected in the B6xD2 intercrosses. Thus, the possibility still remains that in some crosses, preference and anxiety may be related. This argument could be extended from different inbred mouse strain crosses to different species (e.g., rats). However, the data in rats show the same complexity observed in mice. For example, McKinzie and colleagues (2000) have observed that alcohol-preferring (P) rats show significantly greater FPS responses than alcohol nonpreferring (NP) rats. These data suggest that some of the genes associated with alcohol preference also affect FPS, therefore linking a measure of fear conditioning to alcohol preference. However, other rat-research-derived data linking anxiety and alcohol responses are less clear. Hall and colleagues (1998) examined Fawn Hooded (i.e., P rats) and Wistar rat strains and concluded that measures of anxiety were independent of alcohol’s stimulatory and anxiety-reducing effects. McMillen and colleagues (1998) compared the P rats with Wistar rats, the strain from which the P rats were derived. Using a variety of measures, including the elevated plus maze, the authors concluded that anxiety and impulsiveness are not associated with preference. Knapp and colleagues (1997) examined the relationship between emotional state and alcohol intake in several rat lines and strains, using as a measure of emotional state the tendency to emit ultrasonic vocalizations in response to an aversive but nonpainful stimulus (e.g., an air puff). The authors concluded that “the relationship between emotional state and ethanol drinking is complex and cannot be attributed to a simple elevated state of anxiety or emotionality.”

Finally, researchers should not ignore the possibility that the lack of correlation in some studies between preference and fear/anxiety could reflect weaknesses in either the models for alcohol preference or consumption and the models for fear/anxiety. For example, strain or selected line differences in taste or caloric factors could confound differences in preference. However, considerable data (described in part elsewhere in this issue) indicate that this is not the case for both the mouse and rat strains and lines previously discussed. Overall, the data reviewed in this section force researchers to question whether an animal model exists that mimics the high comorbidity of alcohol-related disorders and anxiety and impulsiveness observed in the type II alcoholic.^5^ The current models appear less than ideal. Improvements in the animal models will stem from two sources. One, alcoholism will need to be defined into ever more rigorous and homogeneous phenotypes and the new classifications will need to include informative and easily measured endophenotypes. Two, scientists working with animals will need to be vigorous in building new models that incorporate the new clinical classifications but at the same time maintain ethological perspective.

## Figures and Tables

**Table 1:** Summary of Animal Models^1^

Behavior Modeled	Phenotype	Description
Startle Reactivity	Acoustic Startle Response (ASR) Tactile Startle Response (TSR)	Both the ASR and TSR measure startle reactivity and, in general, show a good correlation to each other. The ASR is elicited with a loud noise; the TSR is generally elicited with an air puff. The response (reflex) is measured by coupling the startle platform to a strain gauge transducer or some similar device. In humans, the startle response is generally measured by the strength of the eye-blink reflex. Habituation to the startle response is abnormal in schizophrenia.
Sensorimotor Gating	Prepulse Inhibition (PPI)	PPI refers to a reduction in the startle response that is observed when a brief (e.g., 20 millisecond) sensory stimulus (the prepulse stimulus) is delivered prior (100 to 1000 milliseconds) to the stimulus that induces the ASR or TSR. The prepulse stimulus inhibits the magnitude of the ASR or TSR. The prepulse can be different from the startle stimulus. In general, treatments that increase brain dopaminergic activity decrease PPI. PPI is reduced in schizophrenia. However, any disorder (e.g., drug abuse) that is likely to involve brain dopamine systems is a candidate to affect PPI.
Associative Processes	Latent Inhibition (LI)	LI refers to the inhibition of a conditioned response (e.g., avoidance of a shock) caused by preexposure to the conditioned stimulus (CS) (e.g., a loud tone). The preexposure phase consists of random exposure to a CS. In the test phase, the CS is presented with an aversive unconditioned stimulus (US) (e.g., a scrambled footshock). Preexposure to the CS delays acquisition of the conditioned avoidance response. Numerous human paradigms are available for measuring LI. LI is reported to be abnormal in schizophrenics (i.e., they more quickly make the CS–US association). In general, the animal data suggest that PPI and LI are measuring different processes.
Conditioned Fear	Fear Potentiated Startle (FPS)	In a typical FPS paradigm, the degree of conditioned fear is reflected by the increased amplitude of the ASR or TSR elicited in the presence of a CS previously paired with an aversive US. In humans, the ASR is enhanced both in anticipation of aversive shock and in association with certain disorders (e.g., posttraumatic stress disorder).
Contextual Fear Conditioning		Contextual fear conditioning is measured by noting the change in locomotor activity (generally bouts of freezing in place) when the animal is placed in the chamber where the CS was paired with an aversive US.
Cued Fear Conditioning		Cued fear conditioning is conceptually similar to FPS. Rather than measuring the change in ASR, the change in locomotor activity is measured after presentation of the CS. To avoid confusing cued fear with contextual fear, researchers must ensure that the test chamber and conditioning chamber are viewed differently.
Unconditioned Fear	Open-Field Activity	The open-field test assesses some aspects of unconditioned fear and anxiety. The standard open field consists of a brightly lit, circular arena with opaque walls. The floor of the open field is scored in a grid. The animal is placed on a small stage in the open field. Outcome is measured by total activity, activity in peripheral versus central grids (i.e., thigmotaxis), rearing, and defecation. High anxiety and emotionality is evidenced by low activity, especially in the central grids, and increased defecation. Automated devices that retain some aspects of the historical open-field apparatus are available.
Light-Dark Transition		The light-dark transition test forces the animal to make a choice between exploring the brightly lit area of the open field or staying in the dark area (the natural tendency). Anxiety-reducing drugs increase exploration in the bright area.
Elevated Plus Maze		The elevated plus maze is similar in concept to the light-dark test except that the animal must choose between exploring a novel environment and the tendency to escape from the elevated open arms of the maze.
Depression	Reserpine-Induced Behaviors	This is one of the earliest models using drugs to mimic a psychiatric disorder. It is based on the clinical observation that 10 to 20 percent of patients administered reserpine (generally for hypertension) report symptoms of depression. In rodents, moderate doses of reserpine induce locomotor depression. The model is no longer used.
Learned Helplessness		Learned helplessness refers to a variety of paradigms in which the animal is exposed to an inescapable aversive stimulus (e.g., a foot shock). Eventually, a certain proportion of the animals will give up and make no attempt to escape. The model has numerous problems but does show predictive validity in that antidepressants and electroconvulsive shock reverse the learned helplessness in susceptible animals.
Behavioral Despair		Conceptually, behavioral despair is related to learned helplessness. In the typical test, the mouse or rat is forced to swim in a tank from which it cannot escape. At the outset, the animals swim vigorously; however, over time some of the animals become immobile, a condition Porsalt (1981) termed behavioral despair.
Psychosis	Chronic Stimulant Treatment	This model builds from the repeated observation that the chronic administration of central nervous system stimulants can induce paranoid psychosis. In animals, chronic stimulant administration will generally induce sensitization to some behaviors (e.g., stereotyped activity) and habituation to others (e.g., exploratory activity).
*N*-methyl-d-aspartate (NMDA) Receptor Antagonists		Clinically, drugs that act by blocking NMDA receptors (i.e., NMDA receptor antagonists), such as phencyclidine (PCP), ketamine, and dizoclipine induce temporary psychotic symptoms and cognitive disturbances somewhat mimicking the negative symptoms of schizophrenia. In animals, the effect of these drugs is generally measured by the temporary increase in locomotor activity.
Psychotomimetic Drugs		Similar in concept to the NMDA receptor antagonist model, this model generally refers to drugs, such as LSD and mescaline, that induce hallucinations, particularly visual hallucinations. Although visual hallucinations are uncommon in schizophrenia and related psychotic disorders, understanding this relatively rare phenotype would be of value. Animal models generally focus on acute drug administration; response is measured as changes in locomotor activity or behavioral disruption.

1 This list of animal models is not intended to be comprehensive but to provide the reader with an exposure to models that have been important in understanding psychiatric disorders.

References to the models are found in the text.

**Table 2:** Relationships Between Alcohol Consumption, Preference, and Acceptance and Measures of Unconditioned and Conditioned Fear in the BXD Recombinant Inbred Series

	Open-Field Activity 3	Fear-Potentiated Startle 4	Fear-Conditioning Contextual 5	Fear-Conditioning Cued 5
Alcohol consumption^1^	0.16	0.12	0.26	−0.09
Alcohol preference^1^	0.24	0.05	0.23	−0.04
Alcohol acceptance (F)^2^	0.11	−0.40	−0.15	−0.09
Alcohol preference (F)^2^	0.42	−0.01	0.21	−0.10
Alcohol acceptance (M)^2^	−0.08	−0.01	0.12	0.26
Alcohol preference (M)^2^	0.37	−0.02	0.30	0.19

NOTE: These results show that no significant correlations exist between the alcohol phenotypes and the fear and anxiety phenotypes.

SOURCES:

1 Phillips et al. 1994 (female mice only);

2 Rodriquez et al. 1994 (male [M] and female [F] mice);

3 Koyner et al. 2000 (male mice only);

4 McCaughran et al. 2000 (male mice only);

5 Owen et al. 1997 (sex not specified).

## Footnotes

2 The degree of startle response is reduced when a brief “warning” stimulus precedes the startle-eliciting stimulus.

3 When some mice are administered low doses of alcohol, their locomotor activity increases. This measure is often used to test for initial sensitivity to alcohol.

4 A recombinant inbred (RI) series is a set of animal strains derived by inbreeding the offspring of two parent strains. Each strain in an RI series has a unique combination of genes from the parent strains, and all animals in a single strain have the same gene combination.

5 The characteristics of the type II alcoholic include impulsiveness, aggressive tendencies, criminality, and other antisocial behaviors.
